# Organisation, staffing and resources of critical care units in Kenya

**DOI:** 10.1371/journal.pone.0284245

**Published:** 2023-07-27

**Authors:** Wambui Mwangi, Ronnie Kaddu, Carolyne Njoki Muiru, Nabukwangwa Simiyu, Vishal Patel, Demet Sulemanji, Dorothy Otieno, Stephen Okelo, Idris Chikophe, Luigi Pisani, Dilanthi Priyadarshani Gamage Dona, Abi Beane, Rashan Haniffa, David Misango, Wangari Waweru-Siika, for the Kenya Critical Care Registry Investigators

**Affiliations:** 1 Department of Anesthesia and Intensive Care, Nyeri County Referral Hospital, Nyeri, Kenya; 2 Kenya Critical Care Registry, Critical Care Society of Kenya, Nairobi, Kenya; 3 Intensive Care Unit, Aga Khan Mombasa Hospital, Mombasa, Kenya; 4 Egerton University Surgery Department, Nakuru Level V ICU, Nakuru, Kenya; 5 Department of Anesthesia and Critical Care, AAR Hospital, Nairobi, Kenya; 6 Department of Anesthesia and Intensive Care, Kisii County Referral Hospital, Kisii, Kenya; 7 Department of Anesthesia and Intensive Care, MP Shah Hospital, Nairobi, Kenya; 8 Department of Anesthesia and Critical Care, Maseno University, Maseno, Kenya; 9 Department of Anesthesia and Critical Care, Kenyatta National Hospital, Nairobi, Kenya; 10 Mahidol Oxford Tropical Research Unit, Bangkok, Thailand; 11 Centre for Inflammation Research, University of Edinburgh, Edinburgh, Scotland; 12 Department of Anesthesia, Aga Khan University, Nairobi, Kenya; Karolinska Institutet, SWEDEN

## Abstract

**Objective:**

To describe the organisation, staffing patterns and resources available in critical care units in Kenya. The secondary objective was to explore variations between units in the public and private sectors.

**Materials and methods:**

An online cross-sectional survey was used to collect data on organisational characteristics (model of care, type of unit, quality- related activities, use of electronic medical records and participation in the national ICU registry), staffing and available resources for monitoring, ventilation and general critical care.

**Results:**

The survey included 60 of 75 identified units (80% response rate), with 43% (n = 23) located in government facilities. A total of 598 critical care beds were reported with a median of 6 beds (interquartile range [IQR] 5–11) per unit, with 26% beds (n = 157) being non functional. The proportion of ICU beds to total hospital beds was 3.8% (IQR 1.9–10.4). Most of the units (80%, n = 48) were mixed/general units with an open model of care (60%, n = 36). Consultants-in-charge were mainly anesthesiologists (69%, n = 37). The nurse-to-bed ratio was predominantly 1:2 with half of the nurses formally trained in critical care. Most units (83%, n = 47) had a dedicated ventilator for each bed, however 63% (n = 39) lacked high flow nasal therapy. While basic multiparametric monitoring was ubiquitous, invasive blood pressure measurement capacity was low (3% of beds, IQR 0–81%), and capnography moderate (31% of beds, IQR 0–77%). Blood gas analysers were widely available (93%, n = 56), with 80% reported as functional. Differences between the public and private sector were narrow.

**Conclusion:**

This study shows an established critical care network in Kenya, in terms of staffing density, availability of basic monitoring and ventilation resources. The public and private sector are equally represented albeit with modest differences. Potential areas for improvement include training, use of invasive blood pressure and functionality of blood gas analysers.

## Introduction

Critical care, also known as intensive care, is a multidisciplinary and inter-professional specialty dedicated to the comprehensive management of patients having, or at risk of developing acute, life-threatening organ dysfunction [1]. The description of the units in which intensive care is provided varies globally and even within a single healthcare system. However, central to the definition of these critical care units are a dedicated geographical area, the ability for continuous monitoring and support of physiological systems and the availability of a specially qualified, interdisciplinary and interprofessional clinical team [1, 2]. These units are an integral part of the health care system irrespective of the health system capacity [1, 2].

Despite the large burden of critical care illness in low and middle income countries (LMICs), most of these countries lack published data on their ICU capacity [7]. While data on critical care capacity is available from LMICs in Asia at multinational or country level, data from African LMICs is reported only for a small proportion of the 54 African countries [3–10]. Landscaping of existing infrastructure, equipment and staffing is crucial in the planning, mobilisation and resource allocation in healthcare systems. This is fundamental in evaluating quality of service provision while identifying priorities in resource allocation, collective procurement and quality improvement [2].

Starting in the 1950s with two beds in the then King George Hospital in Nairobi, critical care in Kenya is a growing specialty with diverse outcomes [9, 10]. A 2014 study showed 130 critical care beds in 21 hospitals across the country. More than half of these beds were located in private or faith based institutions, making this sector an important provider of critical care services in Kenya [8]. As of 2020, Kenya had an estimated 537 ICU beds and 256 ventilators with only 22 of the 47 counties having at least one critical care unit [5]. However, data on the functionality of these units and distribution within the different health sectors is still limited.

In order to gain insight into the current infrastructure and resources of existing critical care units in the country, we performed a landscaping survey of critical care units across the health facilities in the country in both the public and private sector. We specifically aimed at describing the extent and variation of critical care services provision and organisation, including quality improvement activities, in order to flag potential areas for improvement.

## Methods

### Study setting

Kenya, a lower middle income country in East Africa, has a population of 47.6 million with an inter-censal population growth rate of 2.3% [11]. The Kenyan health care provision is divided between the public and private sectors. Government institutions are referred to as “public” while those owned by any other stakeholder(s) are referred to as “private”. The private facilities include both private for profit and private not for profit (PNFP) institutions [12]. In 2013, with the exception of national referral hospitals, management of the public healthcare system was transferred from the national government to each of the 47 county level governments [13].

### Study design

This was an online cross-sectional survey based study, designed to collect facility level data on organisational structure, staffing and resource characteristics. A study tool that has been used previously to enable comprehensive landscaping of ICU services in resource constrained health systems in Asia and Africa was modified to be used in the current study [14].

### Ethics

Ethical regulatory approval for this study was sought prior to study commencement at National level through the National Commission for Science, Technology and Innovation (licence n° NACOSTI/P/21/13484) and through the Ethical committee of Aga Khan University in Nairobi (Ref. 2021/IERC-125). The need for consent was waived by the Ethical committee since only facility level data was to be collected in the survey, with no individual patient data.

### Units surveyed

Focal persons in all the intensive care units (ICU) and high dependency units (HDU) listed in the Critical Care Society of Kenya (CCSK) and the Kenya Society of Anesthesiology (KSA) records were invited to participate in the survey (**S1 Table** lists study collaborators). The survey link was also sent out to CCSK and KSA members in an effort to identify ICUs facilities and units in Kenya that were not included in the existing KSA/CCSK lists. Whenever a hospital had more than one unit, each was considered separately. The contact person, either a senior member of staff working in the identified unit and/or the in charge of the hospital was invited to fill in the required information online. The survey tool was opened on November 1st 2021 and closed on March 8th 2022.

### Survey design

The first draft of the questionnaire was discussed with the Kenya Critical Care Registry members in June 2021 starting from a previously published survey tool in Pakistan [14], with context specific modifications made following members’ feedback. The questionnaire was piloted among the Critical Care Registry members in October 2021. An online landscaping survey was built on Google Forms® in order to digitally collect and aggregate responses. The detailed survey questions are available in the **S1 File.**

### Data collection

The questionnaire was divided into organisational, staffing, resources and service-evaluation categories. **Organisational variables included number of beds in the unit compared to the hospital beds, ICU status (medical, surgical, COVID unit, etc.), model of care and use of quality related interventions.** The model of ICU care was defined pragmatically as follows: ‘open unit’ in cases where the ICU had access to multiple doctors who were free to admit, manage and discharge their patients; a ‘closed unit’ whenever the admission, discharge and referral policies were under the control of the ICU consultant or intensivist only.

Staffing variables included nurse to bed ratio during daytime and night time, the availability of ICU physicians, specialities available for consultation, training status of critical care nurses. Resources variables included number of functioning ventilators and high flow nasal cannula machines, type of oxygen supply, availability of imaging and monitoring equipment, availability of point of care laboratory equipment; availability of infusion and syringe pumps, difficult airway trolley, defibrillator apparatus; availability of electronic medical record; visiting hours policy before and after COVID-19 pandemic.

The survey also sought to capture existing service evaluation activities in the units. Examples included morbidity and mortality meetings, quality improvement programs, root cause analysis meetings and continuous medical education events. The willingness to participate in a National ICU registry was also assessed.

The validity of the responses was not followed up by site-level data quality checks or source document verification. To facilitate survey completion dropdown questions were preferred over free text. No imputation was performed for missing data. In case of incomplete or inconsistent responses, sites were followed up via telephone by the study investigators (WM, DO, WW) in order to maximise survey completion and accuracy of data.

### Statistical analysis

Due to the descriptive nature of this analysis we did not perform a formal sample size calculation. To comply with the secondary objective, the analysis was performed comparing the units pertaining to the public sector versus units hosted in private or PNFP hospitals. The findings are to be considered as exploratory, as no correction for multiple testing was performed. Descriptive data was summarised as medians and interquartile range for continuous variables and as frequencies (percentage) for categorical variables. All percentages are shown to the respective available number for each group, otherwise it was adjusted to availability and denominator separately reported. In the case of normally distributed, continuous variables were compared between units using t–tests. When not considered normally distributed, continuous variables were compared between groups using Mann–Whitney U tests. Categorical variables were compared between groups by chi–square analysis. Data was analysed using appropriate statistical software (STATA/IC for Mac v16.1, StataCorp LP, Texas, USA). The heatmap displaying participating units was made using appropriate licensed software (Infogram, Prezi.inc, Broadway, Oakland, USA).

## Results

### Survey responders

Of 75 units invited to participate in the survey, 60 (80%) units from 55 hospitals in 21 counties responded (**S2 Table**). Of these units, 17 (28%) were in the capital city, Nairobi and most units were located in the south of the country. There were no critical care units identified in 22 out of the 47 counties.

### Organisational characteristics

**Fig 1:**
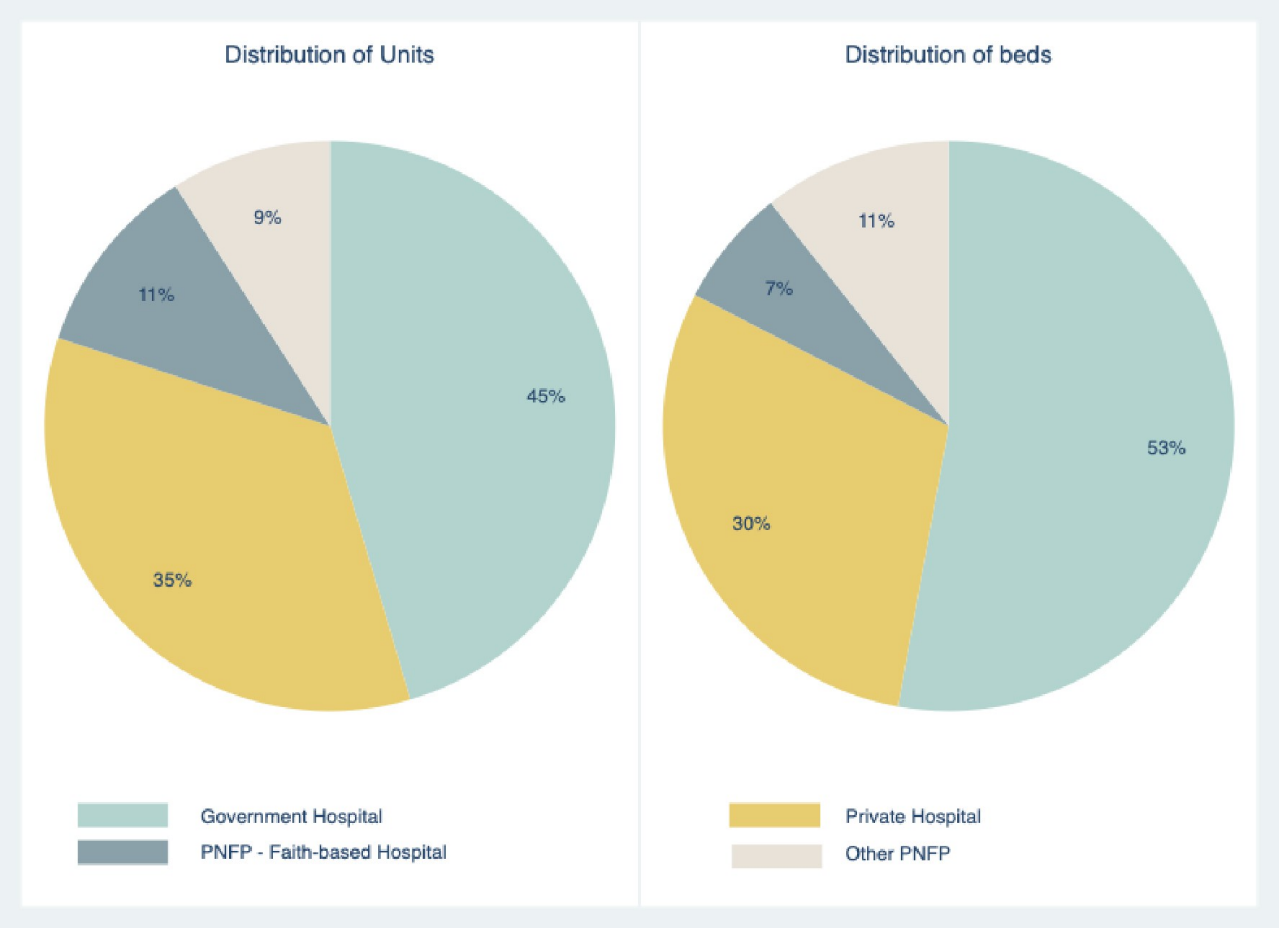
Distribution of critical care units and ICU beds by hospital status. Abbreviations: PNFP, private not for profit.

Detailed information on hospital status, affiliations with educational bodies, hospital capacity, ICU capacity and average admission volumes is provided in **Table 1** and **Fig 1**. Out of a total of 598 ICU beds, 26% (n = 157) were not functional at the time of data collection, with a greater proportion of non-functional beds in the public sector (p = 0.001). Out of the total number of functional beds, 57% (n = 282) were located in private or PNFP hospitals. The median proportion of ICU beds to total hospital beds was overall 3.8% (IQR 1.9–10.4) with a higher proportion reported in the private sector (4.3 [2.8–13.3], p = 0.011). The model of care in Kenyan ICUs was defined as ‘closed’ in only one third of surveyed units (n = 22, 36.7%). Most units (n = 40, 80%) were mixed ICUs in terms of types of patients admitted, and admission of paediatric patients was routinary in 42 (70%) of surveyed facilities.

**Table 1:** Organisational characteristics of participating hospitals and units.

	All units	Public facilities	Private or PNFP facilities	P-value
Hospital-level	n = 55	n = 25 (45.5%)	n = 30 (54.5%)	
**Hospital status, n(%)**				
Private	19 (34.5)	–	19 (34.5)	
Private not for profit	6 (10.9)	–	6 (10.9)	
Other	5 (9.1)	–	5 (9.1)	
**Training programs, n(%)**				
Affiliated with university	10/55 (18.2)	10/25 (40.0)	0	
Affiliated with medical college	21/55 (38.2)	12/25 (48.0)	9/30 (30.0)	0.171
Recognised for internship training for MO/CO/Nurses	35/55 (63.6)	22/25 (88.0)	13/30 (43.3)	0.042*
Recognised for residency training	11/55 (20.0)	8/25 (32.0)	3/30 (10.0)	0.001*
Recognised for ICM training	4/55 (7.2)	3/25 (12.0)	1/30 (3.3)	0.218
None	14/55 (25.5)	1/25 (4.0)	13/30 (43.3)	0.001*
**Number of beds in hospital,**total n	17477	12847	4630	
**Number of beds for each hospital,**	200 (100,350)	324 (200–650)	147 (80–250)	0.0006*
**ICU-LEVEL**	**n = 60**	**n = 26 (43.3%)**	**n = 34 (56.7%)**	
**Total number of beds in ICU**	598	316	282	
Number of beds not in use, (%)	157 (26.3)	126 (39.9)	31 (11.0)	0.000*
Functional beds, (%)	441 (73.7)	190 (59.1)	251 (89.0)	
**Number of total beds for each ICU,** median (IQR)	6 (5–11)	7 (6–12)	6 (4–11)	0.161
**Proportion of ICU beds to total number of Hospital beds,** median (IQR)	3.8 (1.9–10.4)	2.4 (1.2–4.7)	4.3 (2.8–13.3)	0.011*
**Model of care**				0.043*
Open	36 (60.0)	11 (42.3)	25 (73.5)	
Closed	22 (36.7)	14 (53.9)	8 (23.5)	
Semi-closed	1 (1.7)	0 (0)	1 (2.9)	
Not referred	1 (1.7)	1 (3.9)	0 (0)	
**Type of unit**				0.754
Mixed ICU	48 (80.0)	22 (84.6)	26 (76.5)	
Surgical ICU	0 (0)	0 (0)	0 (0)	
Medical ICU	1 (1.7)	0 (0)	1 (2.9)	
Cardiothoracic	2 (3.3)	1(3.9)	1 (2.9)	
High dependency Unit	2 (3.3)	-	2 (5.9)	
COVID ICU	4 (6.7)	2 (7.7)	2 (5.9)	
Other	3 (5.0)	1 (3.9)	2 (5.9)	
**Total admissions past 12 months**				0.882
0–50	12 (20.0)	6 (23.1)	6 (17.7)	
50–100	9 (15.0)	4 (15.4)	5 (14.7)	
100–200	18 (30.0)	8 (30.8)	10 (29.4)	
200–300	5 (8.3)	3 (11.5)	2 (5.9)	
300–400	3 (5.0)	1 (3.9)	2 (5.9)	
> = 400	13 (21.7)	4 (15.4)	9 (26.5)	
**Routine paediatric admissions**	42 (70.0)	19 (73.1)	23 (67.7)	0.649

Data is presented as median (IQR) or n(%).

Abbreviations: MO, Medical Officer; CO, Clinical Officer; ICM, intensive care medicine.

While quality related activities were conducted across most of the units, private units more frequently reported quality improvement programs (p = 0.009) and root cause analysis meetings (p = 0.006; Table 2). Electronic medical records were used in 21 units (36.2%) with a higher proportion in the private sector (p = 0.002). All respondents expressed an interest in participating in the national ICU registry, with current participation reported in 17% of units.

**Table 2:** Quality-related activities, use of electronic medical records and participation in national quality improvement programs.

	All units (n = 60)	Public facilities (n = 26)	Private or PNFP facilities (n = 34)	P value
**Unit Quality activities**				
M&M meetings	47 (78.3)	18 (69.2)	29 (85.3)	0.134
QI programs	30 (50.0)	8 (30.8)	22 (64.7)	0.009*
Root cause analysis meeting	26 (43.3)	6 (23.1)	20 (58.8)	0.006*
CME	49 (81.7)	23 (88.5)	26 (76.5)	0.234
Debriefing sessions	40 (66.7)	16 (61.5)	24 (70.6)	0.461
**Use of electronic medical record**				0.002*
Current use	21 (36.2)	4 (15.4)	17 (53.1)	
Planned	5 (8.6)	1 (3.9)	4 (12.5)	
No	32 (55.20)	21 (80.8)	11 (34.4)	
**Participation in the National ICU registry (current)**	10 (16.7)	4 (15.4)	6 (17.7)	0.816

Numbers are shown as value (%)

Abbreviations: EMR, electronic medical record; QI, quality improvement; M&M, morbidity and mortality; QI, quality improvement; CME, continuous medical education

### Staffing and training

Unit staffing is described in **Table 3**. A consultant in charge was present in 54 (90%) of the units, the primary speciality being mainly Anaesthesia (n = 37, 69%). In 23 (40%) of units the consultant in charge did not have formal training in critical care, with a higher proportion of formally trained consultants being in the private sector (p = 0.047). A total of 19 (31.7%) units reported the absence of a dedicated clinician during daytime, with the number increasing slightly to 24 units (40%) during night time. There were no significant differences in availability of dedicated clinicians between public and private units.

**Table 3:** Unit’s staffing information.

	All units(n = 60)	Public facilities (n = 26)	Private or PNFP facilities (n = 34)	P value
**Consultant in charge, yes**				
Anesthesiologist	37/54 (68.5)	19/23 (82.6)	18/31 (58.1)	0.077
Medicine	9/54 (16.7)	2/23 (8.7)	7/31 (22.6)	0.273
Surgeon	4/54 (7.4)	2/23 (8.7)	2/31 (6.5)	0.756
Other*	7/54 (13.0)	0/23 (0.0)	7/31 (22.6)	
**Formal training in ICM**				0.047*
Yes, <2 years	14/58 (24.1)	6/25 (24.0)	8/33 (24.4)	
Yes, >2 years	21/58 (36.2)	5/25 (20.0)	16/33 (48.5)	
No	23/58 (39.7)	14/25 (56.0)	9/33 (27.3)	
**Clinician dedicated to the unit during the DAY**				
Consultant	8 (13.3)	2 (7.7)	6 (17.7)	0.501
Non-consultant doctor	28 (46.7)	14 (53.9)	14 (41.2)	
Clinical Officer	5 (8.3)	3 (11.5)	2 (5.9)	
None	19 (31.7)	7 (26.9)	12 (35.3)	
**Clinician dedicated to the unit during the NIGHT**				0.343
Consultant	2 (3.3)	0(0.0)	2 (5.9)	
Non-consultant doctor	27 (45.0)	11 (42.3)	16 (47.1)	
Clinical Officer	7 (11.7)	5 (19.2)	2 (5.9)	
None	24 (40.0)	10 (38.5)	14 (41.2)	
**Nurse to bed ratio**				
** during day**				0.080
1:1	17 (28.3)	6 (23.1)	11 (32.4)	
1:2	33 (55.0)	12 (46.2)	21 (61.8)	
1:3	9 (15.0)	7 (26.9)	2 (5.9)	
1:4	0 (0.0)	0 (0.0)	0 (0.0)	
1:5 or more	1 (1.7)	1 (3.9)	0 (0.0)	
** during night**				0.452
1:1	11 (18.3)	3 (11.5)	8 (23.5)	
1:2	36 (60.0)	15 (57.7)	21 (61.8)	
1:3	10 (16.7)	6 (23.1)	4 (11.8)	
1:4	1 (1.7)	1 (3.9)	0 (0.0)	
1:5 or more	2 (3.3)	1 (3.9)	1 (2.9)	
**% of Nurses with formal training in ICM nursing, median (IQR)**	48.9 (83.0–27.9)	52.8 (83.3–40.0)	40.0 (82.6–20.0)	0.097
**Nurse in charge with formal training in ICM nursing**				0.010*
Yes—Higher National diploma	44 (73.3)	24 (92.3)	20 (58.8)	
Yes—Other diploma	7 (11.7)	0 (0.0)	7 (20.6)	
No	9 (15.0)	2 (7.7)	7 (20.6)	
**Nurse assistants present**	30 (50.0)	5 (19.2)	25 (73.5)	<0.001

Data is presented as median (IQR) or n(%)

*Other: Intensivist, Nephrologist, Emergency Medicine Physician.

PFNP, Private not for profit; ICM, Intensive Care Medicine

The nurse to bed ratio during daytime was 1:2 in 33 (55%) of ICUs, with a 1:1 ratio reported by 28.3% (n = 17) of responders. There was no significant difference in the nurse to bed ratio between public and private facilities (p = 0.08). Half of the total number of nurses (IQR 27.9–83.0) and 51 (85%) of the nurses in charge of the units had undergone formal training in critical care nursing, with a higher proportion in the public facilities (p = 0.010). Half of the units (n = 30) did not have nurse aides available to assist in patient care, with aides missing especially in the public sector (p<0.001).

Access to specialist consultation was frequent for all main medical disciplines. However, less than a third of units had access to haematologists and microbiologists (**Table 4**). Nutritionists, pharmacists, physiotherapists, counsellors and psychologists were mainly available on consultation. The public facilities had more nutritionists (p = 0.002) and physiotherapists (p = 0.02) dedicated to the units (**Table 4**).

**Table 4:** Additional staffing information.

	All units (n = 60)	Public facilities (n = 26)	Private or PNFP facilities (n = 34)	P value
**Availability of specialist consultation**				
Anesthesiology	50 (83.3)	21 (80.8)	29 (85.3)	0.733
Physician	56 (93.3)	25 (96.2)	31 (91.2)	0.626
General surgeon	55 (91.7)	24 (92.3)	31 (91.2)	0.875
Obstetric Gynaecologist	54 (90.0)	24 (92.3)	30 (88.2)	0.689
Cardiologist	28 (46.7)	8 (30.8)	20 (58.8)	**0.031**
Neurosurgeon	34 (56.7)	10 (38.5)	24 (70.6)	**0.013**
Nephrologist	28 (46.7)	8 (30.8)	20 (58.8)	**0.031**
Gastroenterologist	26 (43.3)	5 (19.2)	21 (61.8)	**0.001**
Neurologist	18 (30.0)	3 (11.5)	15 (44.1)	**0.006**
Microbiologist	11 (18.3)	4 (15.4)	7 (20.6)	0.606
Urologic surgeon	36 (60.0)	13 (50.0)	23 (67.6)	0.167
Haematologist	17 (28.3)	4 (15.4)	13 (38.2)	0.052
Pathologist	31 (51.7)	13 (50.0)	18 (52.9)	0.821
Orthopaedic surgeon	49 (81.7)	21 (80.8)	28 (82.4)	0.875
Paediatrician	52 (86.7)	23 (88.5)	29 (85.3)	0.721
Cardiothoracic Surgeon	24 (40.0)	7 (26.9)	17 (50.0)	0.071
Respiratory disease specialist	19 (31.7)	4 (15.4)	15 (44.1)	**0.018**
Other*	2 (3.3)	1 (4.2)	1 (2.9)	0.847
**Nutritionist available**				**0.002**
Dedicated to the unit	19 (31.7)	14 (53.9)	5 (14.7)	
Available on consult	36 (60.0)	9 (34.6)	27 (79.4)	
Not available	5 (8.3)	3 (11.5)	2 (5.9)	
**Physiotherapist**				**0.020**
Dedicated to the unit	22 (36.7)	14 (53.9)	8 (23.5)	
Available on consult	37 (61.7)	11 (42.3)	26 (76.5)	
Not available	1(1.7)	1 (3.9)	0	
**Counsellor or psychologist**				0.279
Dedicated to the unit	6 (10.0)	4 (15.4)	2 (5.9)	
Available on consult	43 (71.7)	16 (61.5)	27 (79.4)	
Not available	11 (18.3)	6 (23.1)	5 (14.7)	
**Pharmacist**				0.764
Dedicated to the unit	7 (12.1)	4 (15.4)	3 (9.4)	
Available on consult	41 (70.7)	18 (69.2)	23 (71.9)	
Not available	10 (17.2)	4 (15.4)	6 (18.8)	
**Radiology technician for portable radiology**	46 (76.7)	20 (76.9)	26 (76.5)	0.967

Data is presented as median (IQR) or n(%)

* Other: Psychiatrist, Family medicine physicians

### Equipment resources

The structure of beds and availability of monitoring devices is detailed in **Table 5**. Most beds (IQR 50–100) had a pressure relieving mattress, but with a higher percentage in the private sector (p = 0.011). Nearly 40% (IQR 0–100) of the beds lacked an electric motor for tilting purposes. All of the beds had a multiparametric patient monitor with non-invasive blood pressure monitoring capacity. However, invasive blood pressure monitoring was only available on 2.5% (IQR 3.0–81.3) and capnography on 31% (IQR 0–77.4) of the critical care beds. Equipment for measurement of cardiac output was available in 12% (n = 7) of units.

**Table 5:** Bed structure, monitoring devices and ventilators.

	All units (n = 60)	Public facilities (n = 26)	Private or PNFP facilities (n = 34)	P value
**Bed structure**				
Pressure relieving mattress per unit (%) median % (IQR)	100.0 (50–100)	75.0 (42.9–100)	100.0 (85.7–100)	**0.011**
Beds with electric motor per unit (%) median % (IQR)	60.0 (0–100)	45.9 (0–100)	92.9 (0–100)	0.203
**Monitoring devices**				
Non-invasive Blood pressure (% of beds per unit) median % (IQR)	100 (100–100)	100 (100–100)	100 (100–100)	0.479
Invasive blood pressure per unit (% of beds per unit) median % (IQR)	2.5 (0–81.3)	0 (0–33.3)	31.0 (0–100)	0.094
Capnography per unit (% of beds per unit) median % (IQR)	31.0 (0–77.4)	42.7 (0–100)	26.8 (0–62.5)	0.541
Cardiac output, number of units (%)	7 (11.7)	1 (3.9)	6 (17.7)	0.099
**Blood gas analysis, n(%)**	56 (93.3)	22 (84.6)	34 (100.0)	**0.018**
With Hb measurement	50 (89.3)	20 (90.9)	30 (88.2)	0.752
With lactate measurement	41 (73.2)	17 (77.3)	24 (70.6)	0.581
Functional blood gas analysis	45 (80.4)	12 (54.6)	33 (97.1)	**<0.001**
**Location of BGA machine, n(%)**				**0.017**
Main Hospital laboratory	15 (26.8)	2 (0.1)	13 (38.2)	
Satellite hospital laboratory	2 (3.6)	0 (0)	2 (5.9)	
Another ICU	4 (7.1)	3 (13.6)	1 (2.9)	
In ICU	35 (62.5)	17 (77.3)	18 (52.9)	
**Ventilation devices**	n = 57	n = 26	n = 31	
ICUs with all beds equipped with a ventilator	47/57 (82.5)	22/26 (84.6)	25/31 (80.6)	
With paediatric mode	33/46 (71.7)	17/21 (81.0)	16/25 (64.0)	
**Total number of functional ventilators per unit**	n = 57	n = 26	n = 31	**<0.001**
	7 (12–4)	11 (16,7)	4 (7,3)	
**Number of HFNT machines per unit median (Q3-Q1)**	0 (2–0)	0 (0–0)	0.5 (2–0)	0.056
Units with at least 1 HFNT machine	23 (38.3)	6 (23.1)	17 (50.0)	**0.034**
**Defibrillator**	56 (93.3)	23 (88.5)	33 (97.1)	0.307
**Difficult airway trolley**				0.856
Without percutaneous tracheostomy kit	30 (50.0)	12 (46.2)	18 (52.9)	
With percutaneous tracheostomy kit	5 (8.3)	2 (7.7)	3 (8.8)	
*None*	25 (41.7)	12 (46.2)	13 (38.2)	
**Syringe pumps**				0.468
Yes, 1 per bed	12 (20.0)	4 (15.4)	8 (23.5)	
Yes, 2 per bed	21 (35.0)	10 (38.5)	11 (32.4)	
Yes, 3 or more per bed	11 (18.3)	3 (11.5)	8 (23.5)	
Yes, but not on each patient bed	15 (25.0)	8 (30.8)	7 (20.6)	
No	1 (1.7)	1 (3.9)	0 (0.0)	
**Infusion pumps**				0.141
Yes, 1 per bed	27 (28.3)	10 (38.5)	17 (50.0)	
Yes, 2 per bed	12 (20.0)	5 (19.2)	7 (20.6)	
Yes, 3 or more per bed	3 (5.0)	0 (0.0)	3 (8.8)	
Yes, but not on each patient bed	17 (28.3)	11 (42.3)	6 (17.7)	
No	1 (1.7)	0 (0.0)	1 (2.9)	
**Portable x-ray machine**	45 (75.0)	21 (80.8)	24 (70.6)	0.367
**Ultrasound machine**	44 (73.3)	15 (57.7)	29 (85.3)	**0.017**
Dedicated to the unit	19 (43.2)	10 (66.7)	9 (31.0)	0.322
With a cardiac probe	26 (59.1)	6 (40.0)	20 (69.0)	**0.006**
With a convex probe	32 (72.7)	11 (73.3)	21 (72.4)	0.134
With a linear probe	39 (88.6)	14 (93.3)	25 (86.2)	0.113

Data is presented as median (IQR) or n(%)

*Percentage of unit beds with the monitoring device or technique available.

Abbreviations: IQR, interquartile range; ICU, intensive care unit; HFNT, high flow nasal cannula

Blood gas analysis machines were widely available (n = 56, 93%) with all the private units and 84.6% (n = 22) of public units having a blood gas machine. Almost half of the analyzers (n = 10) were reported not functional in the public sector. An ultrasound machine was available in 44 units (73.3%) with a significantly higher number in the private sector (p = 0.017). Although nearly 26 units (59.1%) reported having an ultrasound machine with a cardiac probe, only one unit reported monitoring cardiac output through echocardiography.

Most units (82.5%, n = 47) reported a ventilator on each available bed. The total number of functional ventilators reported in the country is 570 with a median 7 beds per unit (IQR 4–12). However, there was a higher number of ventilators per unit reported in public ICUs (p<0.01). Most units did not have a functional high flow nasal therapy (HFNT) machine available, with this technique being slightly more available in private facilities (p = 0.034). The main form of humidification was the use of humidity mixture exchange (HME) filters. Only one unit reported not having a syringe pump with 15 units (25%) sharing a device among the beds. Similarly, one unit reported a lack of an infusion pump with 17 units (28.9%) having the device shared between the beds.

### Structural resources and unit operations

Details concerning structural features of the units are detailed in **Table 6** and **S3 Table.** Almost all of the units (n = 56, 93.3%) sampled had a backup generator. A total of 71 isolation rooms were reported with 39 units (68.9%) having at least one isolation room. However, only 21 (29.6%) of these were equipped with a negative pressure system. An oxygen plant was the main source of oxygen for most units (n = 31, 53.3%) with a third (n = 19) of units relying on bedside oxygen cylinders and concentrators. Functional wall suction was available in 68.3% (n = 41) of units while compressed air was available in 36 units (60%). More than half of units reported a closure in the previous year, with the most common reason for suspended operations being infection or need for fumigation. Visiting policy was confined to hospital visiting hours in most units, with little change due to the pandemic (**S3 Table**).

**Table 6:** Structural characteristics.

	All units(n = 60)	Public facilities (n = 26)	Private or PNFP facilities (n = 34)	P value
**Backup generator**	56 (93.3)	23 (88.5)	33 (97.1)	0.186
**Source of oxygen**				
Bedside cylinders	14 (23.3)	4 (15.4)	10 (29.4)	0.203
Bedside oxygen concentrators	5 (8.3)	3 (11.5)	2 (5.9)	0.432
Cylinders in manifold system	16 (26.7)	5 (19.2)	11 (32.3)	0.255
Hospital concentrator plant	31 (53.3)	17 (65.4)	15 (44.1)	0.102
Liquid oxygen tank	18 (30.0)	9 (34.6)	9 (26.5)	0.495
**Wall suction**				
Units	41 (68.3)	19 (73.1)	22 (64.7)	0.490
Beds	389	172	217	
**Piped compressed air**				
Units	36 (60.0)	13 (50.0)	23 (67.7)	0.167
Beds	337	133	204	
**Handwashing facilities**	58 (96.7)	25 (96.2)	33 (97.1)	0.847
**Isolation rooms**	n = 57	n = 25	n = 32	
At least one isolation room	39(68.4)	19(76.0)	20(62.5)	0.277
Number of isolation rooms, median (IQR)	1(2–0)	1(1–1)	1(2–0)	0.939
Total number of isolation rooms	71	25	46	
**Of which Negative pressure rooms**	21/71 (29.6)	5/25 (20.0)	16/46 (34.8)	0.199
**Single use plastic aprons**	49 (81.7)	17 (65.4)	32 (94.1)	**0.004**
**Telephone services**				
Direct landline	39 (6.0)	13 (50.0)	26 (76.5)	**0.033**
Mobile line	17 (28.3)	11 (42.3)	6 (17.7)	**0.036**
Intercom	5 (8.3)	3 (11.5)	2 (5.9)	0.432
**Connection to Internet**				0.073
Yes	41 (68.3)	14 (53.9)	27 (79.4)	
Yes, but it is not functional	8 (13.3)	6 (23.1)	2 (5.9)	
No	11 (18.3)	6 (23.1)	5 (14.7)	
**Closure of unit due to infection reasons** *	16 (26.7)	8 (30.8)	8 (23.5)	0.530
**Closure of unit due to other reasons** *	20 (33.3)	10 (38.5)	10 (29.4)	0.461
**Visiting allowed**	58 (96.7)	24 (92.3)	34 (100.0)	0.100
Other	5 (8.3)	2 (7.7)	3 (8.8)	0.720

*Details on timing of units closure and visiting policy are in the supplement

## Discussion

This survey from critical care units in Kenya identified a substantial yet highly heterogeneous critical care services availability across the country.

A study conducted in 2014 showed a total of 130 ICU beds in Kenya in 21 hospitals, with private facilities having more beds and better equipped than the public facilities [8]. Our findings show an increase in both the number of ICU beds and hospitals with ICUs in the country. The study also found a rather even distribution of critical services between the public and the private sector, with narrow differences in organisation and available resources. Following a needs assessment in 2014, the Kenyan government initiated the managed equipment service (MES) project. The project initiated in 2015 trained users and equipped public hospitals with ICU and other specialised equipment [15]. Despite these investments, the distribution of critical care services in Kenya, like other African countries, remains geographically inhomogeneous with most beds concentrated in the capital city [3, 6, 16]. The lack of a critical care unit in 22 out of 47 Kenyan counties and the limited surge capacity of the country was underlined in a recent article by Barasa et al [5]. These combined findings call for a need assessment for potential expansion of critical care services in Kenya.

The proportion of ICU beds is an evolving feature of the Kenyan critical care system. In 2014, the ICU bed capacity in Kenya was at 0.29 beds/100,000 [4, 8]. Our study estimated an ICU-HDU bed capacity of 1.3 beds/100,000 people. While this can be considered an underestimate since the survey did not manage to capture all the units in the country, it is still a large improvement in comparison to 2014 [4, 8]. This ratio is higher than that in Uganda (0.13 beds/100,000 people), Nigeria (0.2 beds/100,000 people), Ethiopia (0.3 public ICU beds/100,000 people) and Ghana (0.5 beds/100,000 people) [6, 3, 16, 17]. The increase in bed capacity may have resulted from the government investment in 2015 and possibly from the increased demand for ICU beds during the COVID-19 pandemic. However, contemporary data on the number and functionality of ICU beds is pivotal, as a 2015 systematic review showed that more than half of LMICs lacked data on ICU capacity [7]. The 2016 African Surgical Outcomes-2 Trial (ASOS-2 trial) reported the scarcity of critical care resources as a contributor to higher risk of perioperative death in Africa [18]. Although levels of care may differ widely, an increase in ICU capacity will potentially lead to a decrease in the “failure to rescue” phenomenon, i.e. death after a complication, which remains 17 times higher in Africa than high-income countries [19].

Staffing data showed a frequently high nurses to bed ratio, similar to that in Nigeria, Uganda and Ethiopia [3, 6, 17]. The surprisingly high nurse to bed ratio could, as in the Uganda study, be explained by the relatively small number of ICU beds per unit (median of 6 beds per unit) [17]. A high nurse to bed ratio has been associated in some studies with an improvement in patient centred outcomes including achieving a reduction in avoidable harms such as secondary infections, unplanned extubations and ICU delirium [20, 21]. A recent study from Brazil underlined the importance of nurse autonomy rather than staff to bed ratios, a finding that needs confirmation in African settings [22]. Yet, our survey did not contain patient level data and it was not designed to investigate the effect of staffing patterns on patient outcomes or staff performance. The impact of staffing patterns on domains such as infection prevention and control are of particular importance in LMICs where patients face worse infection rates and antibiotic resistance patterns compared to high-income countries [23].

The availability of fully trained intensivists in Kenya, as in many other African countries, remains low in comparison to other middle income countries in South Asia and Latin America [9,24–27] The scarcity of trained ICU consultants, combined with a prevalent open model of care may negatively affect the gains achieved with the national investments in resources and training. The closed model of care with intensivist-led patient management has been found to be associated with better patient outcomes and higher compliance with indicators of ICU care quality [28]. Yet, this model of care was reported in only one third of the units in our study, similar to what was found in Pakistan and Nigeria [3, 6, 16]. Although the open model of care may be mandated by scarcity of intensivists and by resources allocation strategies, the transition to a closed model of care seems an attractive target for the national critical care system.

The availability of basic monitoring devices was ubiquitous while access to more advanced monitoring techniques such as capnography, invasive blood pressure and cardiac output was very low. Capnography is considered a necessity in level 1 units with invasive blood pressure monitoring as a mainstay of level 2 units according to the ICU classification by Marshall et al [1]. The lack of cardiac output monitoring despite availability of ultrasound machines with cardiac probes could be as a result of lack of training on the use of such devices in the country [29].

Invasive mechanical ventilation capacity was extended to a large number of the functional ICU beds, a coverage that may have improved due to the COVID-19 pandemic [30]. The finding that half of blood gas analysis apparatuses in the public sector were reported non-functional may represent an actionable quality improvement target in the patient management process. Blood gas analysis and chest radiographs are fundamental to diagnose conditions such as the acute respiratory distress syndrome (ARDS), that affects one in ten patients entering the ICU [27, 31]. Improved monitoring and imaging techniques in LMICs may aid to tackle the large difference in outcomes still burdening mechanically ventilated patients, as compared to high income countries [32].

Findings on the use of quality improvement related activities was promising. Strategies to improve quality of patient care, especially in limited-resource ICUs, are driven by process management [19]. Protocolised care may improve patient-centred outcomes in Africa, although evidence is still limited [33]. Protocolised care is also advocated to reduce variations in patient outcomes [10] as having a well equipped ICU was shown to be not sufficient to align outcomes in a recent Kenyan study [34]. The recent development of a National Critical Care Registry in Kenya is another step to foster evaluation of care, benchmarking and shared quality improvement initiatives [35].

This study has several limitations. Despite efforts in unit recruitment, the survey coverage was incomplete as some units may have been missed out and others failed to respond. We lack data on the reasons for unwillingness to participate in the survey for the 15 non-responder units. The characteristics of individuals completing the survey were not collected and the validity of the responses was not followed up by site-level data quality checks or source document verification. We also acknowledge that the survey tool lacked information on renal replacement therapy availability, pulse oximetry functionality, electrocardiography and the exact physician to bed ratio, and on maintenance of key ICU equipment. We also did not investigate the reasons for non-functionality of ICU beds to limit survey density and optimise response rate. Data was collected during a period of 5 months during which some changes may have occurred in the participating units.

### Recommendations

This study shows a well established critical care network in Kenya, with capacity for providing monitoring and ventilation for critically ill patients in both the public and private sector. The survey highlights the ongoing need for focused critical care training of intensive care specialists and nurses. Furthermore the maintenance and quality control of point of care testing equipment remains to be improved. The recently established Kenya Critical Care Registry [35], now including 76 beds across 10 ICUs, provides capacity for near real time data for service evaluation, research and quality improvement initiatives that can provide mechanisms for future nation-wide improvement processes.

## Supporting information

S1 Table Collaborators list. (DOCX)

S2 Table Number of units for each county with at least one responder. (DOCX)

S3 Table Additional structural characteristics. (DOCX)

S1 File Complete list of survey questions. (PDF)
