# Supplementary material for: Organisation, staffing and resources of critical care units in Kenya
Source: PLoS One. 2023 Jul 27;18(7):e0284245. doi: 10.1371/journal.pone.0284245 (PMC10374136; doi:10.1371/journal.pone.0284245)
Supplement: S1 Table — (DOCX) [file pone.0284245.s001.docx]

# **S1 Table. Collaborators list**

| **Name and surname** | **e-mail** |
| --- | --- |
| Kirwa Elisha | kirwaz@gmail.com |
| Wangui Manguyu | Kui.manguyu@gmail,com |
| Josephat Kerema | senorkerema@gmail.com |
| Christopher Kariuki | drkariuki2013@gmail.com |
| Benson Ngari | ngaribenson@gmail.com |
| Matthew Koech | koechkm@gmail.com |
| Vincent Mwenda | vinnmwe13@gmail.com |
| Dan Ndiwa | dakips@gmail.com |
| Sarah Okiya | sarahokiya@yahoo.com |
| Koka Patience | kokapatience@gmail.com |
| Faizan Yaseen | Dr.faizanmd@gmail.com |
| Linda Nguu | lindanguu@gmail.com |
| Violet Awour | awuoromondi.ao@gmail.com |
| Amos Mungai | amo2007ke@yahoo.com |
| Linda Tunje | drlindatunje@gmail.com |
| Alphonce Mwendwa | alphmwendwa@gmail.com |
| Mugambi Ambutu | ambutujoe@gmail.com |
| Peter Murimi |  |
| Amina Daudi | aminadaud55@gmail.com |
| Thomas Kabanya | tommkabanya2@gmail.com |
| Lucy Miriti | Kmiritil@gmail.com |
| Seth Ritho | smwirabua@gmail.com |
| Joan-Nelly Rima | rimajoan8@gmail.com |
| Rosemary Mwangi | rosemarymwangi7@gmail.com |
| Catherine Njenga | cnjenga94@gmail.com |
| Mary Muchina | marymuchina09@gmail.com |
| Tarcisio Wachira | tarcisiowachira87@gmail.com |
| Martin Makau |  |
| John Wekhuyi | johnwekhuyi@yahoo.com |
| Mercy Gakuanyi |  |
| Teddy Thaddeus | [teddythaddeus102@gmail.com](mailto:teddythaddeus102@gmail.com) |
| Patricia Wangeci | [janelpatricia5@gmail.com](mailto:janelpatricia5@gmail.com) |
| Selina Mutuku | [selina.mutuku@akhskenya.org](mailto:selina.mutuku@akhskenya.org) |
| Thomas Kabanya | [tommkabanya2@gmail.com](mailto:tommkabanya2@gmail.com) |
| Annastacia Kioko | [annexiekioko56@gmail.com](mailto:annexiekioko56@gmail.com) |
| Peter Mburu | [pitkama12@gmail.com](mailto:pitkama12@gmail.com) |
