# Supplementary material for: Organisation, staffing and resources of critical care units in Kenya
Source: PLoS One. 2023 Jul 27;18(7):e0284245. doi: 10.1371/journal.pone.0284245 (PMC10374136; doi:10.1371/journal.pone.0284245)
Supplement: S2 Table — (DOCX) [file pone.0284245.s002.docx]

# **S2 Table. Number of units for each county with at least one responder**

| **Country** | **All** | **Public** | **Private or PNFP** |
| --- | --- | --- | --- |
| Nairobi | 17 | 5 | 12 |
| Nyeri | 6 | 3 | 3 |
| Uasin Gishu | 6 | 1 | 5 |
| Kiambu | 5 | 3 | 2 |
| Machakos | 3 | 1 | 2 |
| Mombasa | 3 | 1 | 2 |
| Bungoma | 2 | 1 | 1 |
| Embu | 2 | 1 | 1 |
| Kisumu | 2 | 1 | 1 |
| Meru | 2 | 1 | 1 |
| Tharaka-Nithi | 2 | - | 2 |
| Garissa | 1 | 1 | - |
| Kakamega | 1 | 1 | - |
| Kirinyaga | 1 | - | 1 |
| Kisii | 1 | 1 | - |
| Laikipia | 1 | - | 1 |
| Makueni | 1 | 1 | - |
| Mandera | 1 | 1 | - |
| Murang'a | 1 | 1 | - |
| Nakuru | 1 | 1 | - |
| Narok | 1 | 1 | - |
