# Supplementary material for: Organisation, staffing and resources of critical care units in Kenya
Source: PLoS One. 2023 Jul 27;18(7):e0284245. doi: 10.1371/journal.pone.0284245 (PMC10374136; doi:10.1371/journal.pone.0284245)
Supplement: S3 Table — (DOCX) [file pone.0284245.s003.docx]

# **S3 Table. Additional structural characteristics**

|  | **All units**  **Number (%)** | **Public facilities** | **Private or PNFP facilities** | **P value** |
| --- | --- | --- | --- | --- |
| **ICU-LEVEL** | **n=60** | **n=26 (43.3%)** | **n=34 (56.7%)** |  |
| **Visitors policy before COVID** | *n=58* | *n=24* | *n=34* | 0.827 |
| Anytime | 3 (5.2) | 1 (4.2) | 2 (5.9) |  |
| Only during hospital visiting hours | 36 (62.1) | 16 (66.7) | 20 (58.8 |  |
| Hours specified by ICU team | 19 (32.8) | 7 (29.2) | 12 (35.3) |  |
| **Visitors policy currently** |  |  |  | 0.494 |
| Anytime | 2 (3.3) | 1 (3.9) | 1 (2.9) |  |
| Only during hospital visiting hours | 33 (55.0) | 15 (57.7) | 18 (52.9) |  |
| Hours specified by ICU team | 23 (38.3) | 8 (30.8) | 15 (44.1) |  |
| No visits allowed | 1 (1.7) | 1 (3.9) | - |  |
| Limited to immediate family members | 1 (1.7) | 1 (3.9) | - |  |
| **Closure of unit due to infection related issue** | *n=16* | *n=8* | *n=8* | 0.543 |
| Current closure | - | - | - |  |
| Last week | - | - | - |  |
| Last month | 4 (25.0) | 1 (12.5) | 3 (37.5) |  |
| Earlier this year | 7 (43.8) | 4 (50.0) | 3 (37.5) |  |
| Last year | 4 (25.0) | 3 (37.5) | 1 (12.5) |  |
| Other | 1 (6.3) | - | 1 (12.5) |  |
| **Closure of unit due to non-infectious issue** | *n=20* | *n=10* | *n=10* | 0.547 |
| Current closure | 1 (5.0) | 1 (10.0) | - |  |
| Last week | 1 (5.0) | - | 1 (10.0) |  |
| Last month | 2 (10.0) | - | 2 (20.0) |  |
| Earlier this year | 7 (35.0) | 3 (30.0) | 4 (40.0) |  |
| Last year | 5 (25.0) | 3 (30.0) | 2 (20.0) |  |
| Other | 4 (20.0) | - | 1 (10.0) |  |
| **Reasons for unit closure** | *n=20* | *n=10* | *n=10* | 0.187 |
| Fumigation | 3 (15.0) | - | 3 (30.0) |  |
| Renovations | 6 (30.0) | 3 (30.0) | 3 (30.0) |  |
| Oxygen failure | 2 (10.0) | 1 (10.0) | 1 (10.0) |  |
| Lack of staff or strike | 1 (5.0) | 1 (10.0) | - |  |
| No admissions | 5 (25.0) | 2 (20.0) | 3 (30.0) |  |
| Other | 3 (15.0) | 3(30.0) | - |  |
