# Supplementary material for: Organisation, staffing and resources of critical care units in Kenya
Source: PLoS One. 2023 Jul 27;18(7):e0284245. doi: 10.1371/journal.pone.0284245 (PMC10374136; doi:10.1371/journal.pone.0284245)
Supplement: S1 File — (PDF) [file pone.0284245.s004.pdf]

Kenya ICU and HDU Landscaping Survey

Dear Colleague,

This survey aims to map intensive and high dependency care in the country.

This initiative is promoted by the Critical Care Society of Kenya (CCSK) and will contribute to the understanding and improvement of critical care services in Kenya. This survey will take 10-12 minutes of your time to fill.

IF YOUR INSTITUTION HAS MORE THAN 1 UNIT, PLEASE FILL INFORMATION FOR EACH UNIT ON A SEPARATE FORM.

The information generated will inform on existing ICU and HDU resources in the country. However, the identity of individual units submitting information will not be revealed and only aggregate/summary data will be reported. .

We appreciate and acknowledge your contribution and look forward to collaborating together.

For any question please contact Ms. Dorothy Otieno at CCSK Tel. +254 706707508

Best Regards,

Dr. Wambui Mwangi
Prof. Wangari Siika

* Required

1. Email *

____

Hospital Location

2. Name of Hospital *

____

3. In which COUNTRY is the hospital? *

Mark only one oval.

- ( ) Kenya
- ( ) Ethiopia *Skip to question 5*
- ( ) Namibia *Skip to question 5*
- ( ) Sierra Leone *Skip to question 5*
- ( ) South Sudan *Skip to question 5*
- ( ) Tanzania *Skip to question 5*
- ( ) Uganda *Skip to question 5*

County (Kenya)

4. In which COUNTY is the hospital located?

Mark only one oval.

- Mombasa
- Kwale
- Kilifi
- Tana River
- Lamu
- Taita -Taveta
- Garissa
- Wajir
- Mandera
- Marsabit
- Isiolo
- Meru
- Tharaka-Nithi
- Embu
- Kitui
- Machakos
- Makueni
- Nyandarua
- Nyeri
- Kirinyaga
- Murang'a
- Kiambu
- Turkana
- West pokot
- Samburu
- Trans-Nzoia
- Uasin Gishu
- Elgeyo-Marakwet
- Nandi
- Baringo
- Laikipia
- Nakuru

- Narok
- Kajiado
- Kericho
- Bomet
- Kakamega
- Vihiga
- Bungoma
- Busia
- Siaya
- Kisumu
- Homa Bay
- Migori
- Kisii
- Nyamira
- Nairobi

Hospital Details

5. Hospital status *

Mark only one oval.

- Government Hospital
- Private Hospital
- Private not for profit (PNFP) - Faith based Hospital
- Other Private not for profit (PNFP) Hospital

6. What affiliations or training programs does the hospital have? *

Check all that apply.

- [ ] Affiliated with a university (undergraduate training)
- [ ] Affiliated with a medical college
- [ ] Recognised by professional body for residency training (post graduate)
- [ ] Recognised by professional body for Intensive Care Medicine training
- [ ] Recognised by professional body for internship training for MO/CO/Nurses
- [ ] None of the above

Other: [ ] ______________________

7. What is the TOTAL BED capacity in the hospital?

____

Contact Details

8. NAME of person giving information

____

9. PHONE NUMBER of person giving information (for data clarification)

____

10. What is the DESIGNATION of the person giving information?

Check all that apply.

- [ ] Consultant ICU
- [ ] Fellow ICU
- [ ] Resident doctor
- [ ] Medical officer
- [ ] Senior House Officer (SHO)
- [ ] Nursing officer incharge
- [ ] Nurse
- [ ] Registrar

Unit Details

11. What TYPE of hospital unit is the unit?

Mark only one oval.

- Mixed ICU
- HDU
- COVID ICU
- Surgical ICU
- Medical ICU
- Cardiothoracic ICU
- PICU
- NICU
- Other: ____________________

12. What is the TOTAL NUMBER of beds available in the unit?

____

13. How many beds are CURRENTLY NOT IN USE due to shortage of staff or supplies (or any other reason)?

____

14. What is the approximate number of TOTAL ADMISSIONS into the unit in the past 12 months?

Mark only one oval.

- 0-50
- 50-100
- 100-200
- 200-300
- 300-400
- >400

15. Does the unit routinely admit PEDIATRIC patients?

Mark only one oval.

- Yes
- No
- Other: ____________________

16. What is the description of MODEL OF CARE in the unit?

Mark only one oval.

- Open unit (ICU has access to multiple doctors who are free to admit, manage and discharge their patients)
- Closed unit (admission, discharge and referral policies are under the control of Consultant of ICU or Intensivist only)
- Other: ____________________

17. Is there a CONSULTANT IN CHARGE of the unit?

Mark only one oval.

- Yes
- No

18. What is the PRIMARY SPECIALITY of the Consultant in charge of the unit?

Check all that apply.

- [ ] Anaesthesiologist
- [ ] Cardiologist
- [ ] Pulmonologist
- [ ] Emergency Medicine Physician
- [ ] Surgeon
- [ ] Nephrologist
- [ ] Medicine
- [ ] Not applicable

Other: [ ] ____________________

19. Has the consultant in charge of the unit undergone FORMAL TRAINING in a Critical Care Medicine training program?

Mark only one oval.

- Yes < 2 years
- Yes > 2 years
- No

20. Which of the following specialists are available in the hospital for CONSULTATION? (Tick all that apply)

Check all that apply.

- Anaesthesiologist
- General physician
- General surgeon
- Obstetric-gynaecologist
- Cardiothoracic Surgeon
- Neurosurgeon
- Cardiologist
- Nephrologist
- Gastroenterologist(Emergency endoscopies)
- Neurologist
- Microbiologist
- Respiratory disease specialist
- Urologic surgeon
- Haematologist
- Pathologist
- Orthopaedic surgeon
- Paediatrician
- Option 18

Other: ______________________

21. Is there a clinician specifically allocated to the unit (with no other hospital duties) during the DAY?

Mark only one oval.

- Yes - consultant doctor
- Yes - non consultant doctor (e.g. Medical officer)
- Yes - clinical officer
- No

22. Is there a clinician specifically allocated to the unit (with no other hospital duties) in the NIGHT?

Mark only one oval.

- Yes - consultant doctor
- Yes - non consultant doctor (e.g. Medical officer)
- Yes - clinical officer
- No

23. What is the TOTAL NUMBER of nurses in the unit?

____

24. What is the nurse to bed ratio during the DAY time?

Mark only one oval.

- 1 nurse for 1 bed
- 1 nurse for 2 beds
- 1 nurse for 3 beds
- 1 nurse for 4 beds
- 1 nurse for 5 or more beds

25. What is the nurse to bed ratio during the NIGHT time?

Mark only one oval.

- 1 nurse for 1 bed
- 1 nurse for 2 beds
- 1 nurse for 3 beds
- 1 nurse for 4 beds
- 1 nurse for 5 or more beds

26. What proportion of nurses in the unit have undergone FORMAL TRAINING in critical care nursing?

Mark only one oval.

- None
- <25%
- 25-49%
- 50-75%
- >75%
- 100%

27. Has the nurse in charge of the unit undergone FORMAL TRAINING in critical care nursing?

Mark only one oval.

- Yes - Higher National diploma
- Yes - Other diploma
- No

28. Are there NURSE ASSISTANTS/ AIDES in the unit?

Mark only one oval.

- Yes
- No

29. If yes, how many nurse assistants/aides are in the unit? *

Mark only one oval.

- 1
- 2
- 3
- 4
- 5
- Other: ____________________

30. Is there a NUTRITIONIST in the unit?

Mark only one oval.

- Yes, dedicated to the unit
- Yes, available on consult
- No

31. Is there a PHYSIOTHERAPIST available in the unit?

Mark only one oval.

- Yes - dedicated to the unit
- Yes - available on consult
- No

32. Is there a COUNSELLOR available in the unit?

Mark only one oval.

- Yes - dedicated to the unit
- Yes- available on consult
- No

33. Is there a RADIOGRAPHER available for portable radiology services?

Mark only one oval.

- Yes
- No

Unit Resources

34. Does the unit have a BACK-UP GENERATOR?

Mark only one oval.

- Yes
- No

35. When was the back-up generator LAST used?

Mark only one oval.

- Today
- Yesterday
- Last week
- Last month
- Not known

36. How many ISOLATION ROOMS does the unit have?

Mark only one oval.

- none
- 1
- 2
- 3
- more than 3
- Other: ______________________

37. How many of the isolation rooms are NEGATIVE AIR PRESSURE rooms?

Mark only one oval.

- None
- 1
- 2
- 3
- more than 3
- All

38. What is the SOURCE OF OXYGEN for the unit?

Check all that apply.

- [ ] Cylinders on the bedside
- [ ] Cylinders in a manifold system
- [ ] Oxygen concentrators
- [ ] Plant/liquid oxygen tank

Other: [ ] ______________________

39. How many beds have functional WALL SUCTION?

Mark only one oval.

- None
- Some (<50%) of the beds
- Most(>50% but not all) of the beds
- All of the beds

40. How many beds have piped MEDICAL AIR?

Mark only one oval.

- None
- Some of the beds
- Most of the beds
- All of the beds

41. How many beds have a functional PATIENT MONITOR?

Mark only one oval.

- None
- Some of the beds
- Most of the beds
- All of the beds

42. How many beds have NON-INVASIVE BLOOD PRESSURE monitoring?

Mark only one oval.

- None
- Some of the beds
- Most of the beds
- All of the beds

43. How many beds have INVASIVE BLOOD PRESURE monitoring?

Mark only one oval.

- None
- Some of the beds
- Most of the beds
- All of the beds

44. How beds have functional CAPNOGRAPHY?

Mark only one oval.

- None
- Some of the beds
- Most of the beds
- All of the beds

45. If the unit is an ICU, do all the beds have a functional VENTILATOR?

Mark only one oval.

- Yes
- No

46. If the unit is a HDU, can a patient be VENTILATED in the unit?

Mark only one oval.

- Yes
- No

47. Total number of functioning mechanical ventilators in the unit

____

48. Is there a PEDIATRIC mode in ALL the ventilators?

Mark only one oval.

- Yes
- No

49. Is HUMIDIFICATION for ventilators available?

Check all that apply.

- [ ] Yes - active /electric humidifiers
- [ ] Yes - Heat moisture exchangers (HME)
- [ ] No

50. How many High-Flow Nasal Cannula (HFNC) machines are available?

Mark only one oval.

- None
- 1
- 2
- 3
- 4
- 5
- More than 5

51. How many beds have PRESSURE RELIEVING devices or mattress (e.g. Ripple mattress)?

Mark only one oval.

- None
- Some of the beds
- Most of the beds
- All of the beds
- Other: ______________________

52. How many beds have an electric motor e.g. can perform a passive leg raise manouver (PLR)?

Mark only one oval.

- None
- Some of the beds
- Most of the beds
- All of the beds
- Other: ____________________

53. Is there equipment available to measure CARDIAC OUTPUT?

Mark only one oval.

- Yes
- No

54. If Yes- what type of equipment?

____

55. Are there functional SYRINGE PUMPS available in the unit?

Mark only one oval.

- Yes, 1 per patient
- Yes, 2 per patient
- Yes, 3 or more per patient
- NO functioning syringe pumps available

56. Are there functional INFUSION PUMPS available in the unit?

Mark only one oval.

- Yes 1 per patient
- Yes 2 per patient
- Yes 3 or more per patient
- NO fluid pumps are available

57. Is there a DIFFICULT/SPECIALIZED AIRWAY trolley available in the unit?

Mark only one oval.

- Yes
- No

58. Is there a BLOOD GAS ANALYSIS (BGA) machine in the hospital? *

Mark only one oval.

- Yes
- No

59. Where is the BGA machine located?

Mark only one oval.

- Main hospital laboratory
- Satellite hospital laboratory
- Operation Theatre
- Another ICU
- Within ICU

60. Is the BGA machine currently functional?

Mark only one oval.

- Yes
- No
- Other: ____________

61. Does the machine measure HEMOGLOBIN (Hb)?

Mark only one oval.

- Yes
- No
- Other: ____________

62. Does the machine measure LACTATE?

Mark only one oval.

- Yes
- No

63. Is there a PORTABLE X-RAY machine available for use in the unit?

Mark only one oval.

- Yes - in the unit
- Yes - shared with other departments
- No

64. Is there a functional DEFIBRILLATOR in the unit?

Mark only one oval.

- Yes
- No
- Other: ____________

65. Is there a PORTABLE ULTRASOUND machine available for use in the unit?

Mark only one oval.

- Yes - the machine is in the unit
- Yes- shared with other departments
- No
- Other: ____________

66. Which of the following PROBES does the portable ultrasound machine have?

Check all that apply.

- [ ] Cardiac (sector)
- [ ] Convex (abdominal)
- [ ] Linear probe

Other: [ ] ____________

67. Is LUNG ULTRASOUND routinely performed in the unit?

Mark only one oval.

- Yes
- No

68. Are there facilities for handwashing within the unit?

Mark only one oval.

- Yes
- No

69. Are single use plastic aprons available for patient contact in the unit?

Mark only one oval.

- Yes
- No

70. Does the unit have TELEPHONE services?

Check all that apply.

- Direct Landline
- Mobile phone
- Intercom
- Other: _______________

71. Is the unit connected to the INTERNET?

Mark only one oval.

- Yes
- No

Unit Operations

72. Has the unit ever been closed due to an infection related issue?

Mark only one oval.

- Never
- Currently
- Last week
- Last month
- Earlier this year
- Last year
- Other: ____________

73. Has the unit ever been closed due a reason NOT related to infection?

Mark only one oval.

- Never
- Currently
- Last week
- Last month
- Earlier this year
- Last year

74. What was the reason for closing the unit?

____

75. Are relatives/visitors allowed in the unit?

Mark only one oval.

- Yes
- No

76. Before COVID, when were relatives/visitors allowed into the unit?

Mark only one oval.

- Anytime
- Only during hospital visiting time
- Only during timings specified by the ICU team
- Other: ______________________

77. Currently, when are relatives/visitors allowed into the unit?

Mark only one oval.

- Anytime
- Only during hospital visiting time
- Only during timings specified by the ICU team
- Never allowed
- Other: ______________________

78. Which of the following activities are conducted in the unit? *

Check all that apply.

- [ ] Morbidity and mortality meetings
- [ ] Quality improvement programmes
- [ ] Root cause analysis meeting
- [ ] Continuous Medical Education (CME)
- [ ] Debriefing sessions
- [ ] Other (please describe)

Other: [ ] ______________________

79. Have you heard or know of the KENYA ICU REGISTRY?

Mark only one oval.

- ( ) Yes
- ( ) No
- ( ) Other: ____________________

80. Would you be interested in registering the unit in the Kenya ICU registry?

Mark only one oval.

- ( ) Yes
- ( ) No
- ( ) I would like to know more about the registry first
- ( ) Not applicable (already registered)
- ( ) Other: ____________________

This content is neither created nor endorsed by Google.

Google Forms
